# Deterministic Lateral Displacement (DLD) Analysis Tool Utilizing Machine Learning towards High-Throughput Separation

**DOI:** 10.3390/mi13050661

**Published:** 2022-04-23

**Authors:** Eric Gioe, Mohammed Raihan Uddin, Jong-Hoon Kim, Xiaolin Chen

**Affiliations:** School of Engineering and Computer Science, Washington State University Vancouver, 14204 NE Salmon Creek Ave, Vancouver, WA 98686, USA; eric.gioe@wsu.edu (E.G.); m.uddin1@wsu.edu (M.R.U.); jh.kim@wsu.edu (J.-H.K.)

**Keywords:** deterministic lateral displacement, machine vision, machine learning, high throughput, separation and purification, analysis automation

## Abstract

Deterministic lateral displacement (DLD) is a microfluidic method for the continuous separation of particles based on their size. There is growing interest in using DLD for harvesting circulating tumor cells from blood for further assays due to its low cost and robustness. While DLD is a powerful tool and development of high-throughput DLD separation devices holds great promise in cancer diagnostics and therapeutics, much of the experimental data analysis in DLD research still relies on error-prone and time-consuming manual processes. There is a strong need to automate data analysis in microfluidic devices to reduce human errors and the manual processing time. In this work, a reliable particle detection method is developed as the basis for the DLD separation analysis. Python and its available packages are used for machine vision techniques, along with existing identification methods and machine learning models. Three machine learning techniques are implemented and compared in the determination of the DLD separation mode. The program provides a significant reduction in video analysis time in DLD separation, achieving an overall particle detection accuracy of 97.86% with an average computation time of 25.274 s.

## 1. Introduction

In 2004, Huang et al. [1] developed a method to separate particles in a solution based on their size through mechanical methods, known as deterministic lateral displacement (DLD). DLD was created to improve upon the separation techniques, such as hydrodynamic chromatography, which had limited resolution, or devices dependent upon diffusion for the primary separation method [1]. The DLD system has many advantages compared to other microfluidic particle separation techniques including simplicity, low cost, a unique flow field for manipulating cells, superior resolution, and a low degree of clogging [2]. For the original DLD design presented by Huang et al. [1], there was a significant decrease in process time for DNA separation of bacterial chromosomes, only taking ten minutes compared to the hours it would take for more traditional techniques. Since DLD is mechanically deterministic and continuous, the devices could easily be scaled into a larger automated system that fully analyzes a sample [3]. DLD is often used to filter the relatively smaller particles from the larger particles in the solution. One of the more popular types of media for filtration is human blood. The idea is to filter and sort out circulating tumor cells from the smaller red blood cells and other blood components. Circulating tumor cells are approximately 10–20 µm in diameter, whereas the red blood cells are approximately 6 µm in diameter; other cells, such as leukocytes and lymphocytes, are approximately 5–10 µm in diameter; platelets are around 10 µm in diameter [4,5,6]. The extreme rarity of CTCs in the peripheral blood (a few CTCs per mL of blood [7,8]) makes CTC detection and separation from blood cells a difficult task. Consequently, it is necessary to carefully design a DLD device with an optimal critical diameter to filter a large portion of the circulating tumor cells solely by size. This means that the DLD should be carefully designed with either an optimal critical diameter or a range of critical diameters. Much of the early work in DLD was conducted in the very low Reynolds number (*Re*). However, this low-throughput flow is not conducive to the level needed for rapid analysis and testing of devices. Dincau et al. [3] explored vortex formation in DLD with circular pillars in high-throughput environments, while mitigating vortex formation later through the use of airfoil-shaped pillars [9]. When DLD is high-throughput or high Re flow, there is a shift in particle trajectory due to the transformation of the streamlines [3]. In other words, the intended critical diameter of the device no longer applies in the same manner. Additionally, the expected DLD mode will change as the flow rate increases. With the change to high-throughput DLD experimentation, a large amount of data is generated and current research with DLD devices often requires time-consuming and error-prone manual analysis of the resulting particle separation. Depending upon the device and the parameters of the experiment, the analysis can consume many hours of the researchers’ time, which could be better spent designing new devices or creating models through simulations that may assist in the design of the device. Additionally, in order to achieve efficient high-throughput operation, a high concentration of sample is used, and manual analysis time increases, so there is a need for an automated system for device analysis.

To the best of our knowledge, there are no prevalent examples of automated analysis of experimental DLD devices, except for a portable DLD solution from Salafi et al. [10]. The algorithm for the portable DLD was for very low Re flow and was incapable of differentiating any possible repeated particle detections. The observation window for the model was only a single line of pixels, which could easily miss the particles if the flow was in a high Re environment and was not properly set. This design was due to the difficulties with double-counting particles in past iterations.

In addition, there were examples of automated analysis including machine learning or deep learning with other microfluidic techniques such as flow cytometry. Heo et al. [11] presented a multiple-object-tracking solution to obtain single-cell images from a flow with multiple cells by utilizing convolutional neural networks. While tracking the particles through the flow, the algorithm determined the best image of each individual particle, cropped an image of each cell, and then identified the cell type. Another example was the protocol presented for the Intelligent Image Activated Cell Sorting (iICAS) system created by Isozaki et al. [12] that was capable of real-time cell sorting and identification in addition to population-level and cell-level analysis. The algorithm used in the system relied on machine vision and image processing techniques from the OpenCV library and two deep convolutional neural networks, constructed on the TensorFlow and Keras frameworks. The neural networks were trained on over 2000 images of each type and successfully captured images and identified the cells in less than 32 ms [7].

Chu et al. [8] reported a microfluidic device that utilized machine learning for automated manufacturing at high-throughput levels of encapsulated objects. The need for automation of the process was due to the amount of data generated at high-throughput levels, which would require a large number of trained operators to oversee the microencapsulation process. A convolutional neural network was trained on 6.7 million images for the differentiation of four possible states: dripping, jetting, rupturing, and wetting [13]. The dataset was imbalanced for the images for each of the states, with 80% of the images consisting of the desired state; this was reflected in the accuracy of identifying the encapsulation state, with higher accuracy for the states with more images. The highest performing automation occurred when the neural network was trained for the longest time, i.e., 45 h, on the entire dataset of images.

**Figure 1:**
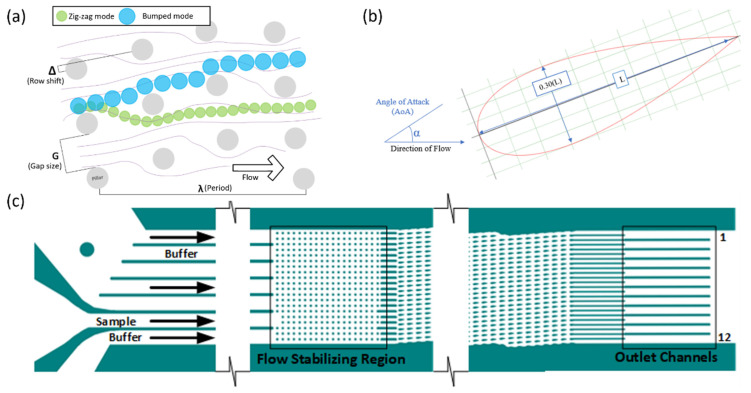
(**a**) The DLD modes “zig-zag” (green) and “bumped” (blue) occur depending upon the size of the particle relative to the critical diameter of the device, which is calculated based the parameters of the device, such as gap size (G), row shift (Δ), and period (λ). (**b**) NACA 0030 Airfoil with a negative angle of attack. (**c**) The DLD device used consisted of 3 inputs: one sample input and two buffer inputs. There was a flow stabilizing region of cylindrical pillars and the displacement or pillar region of airfoil-shaped pillars. There were 12 outlet channels that were the focus of the observation.

The objective of this research paper is to create a software tool that automates the process of observing and characterizing the flow of particles through high-throughput experimental DLD devices to minimize the time required for analysis. To achieve this, a basis of accurately counting the particles with minimal error is established with influence from a variety of works like those previously mentioned. A particle size similar to circulating tumor cells and other blood components is used for testing. Machine learning is employed to predict the DLD mode, including either zig-zag, mixed, or bumped modes, as shown in Figure 1a. The training dataset for the machine learning is built from the particle detection results of the program. In addition, tools to organize machine learning training data, the storage of machine learning models, and the testing of the machine learning models are developed.

## 2. Materials and Methods

### 2.1. Device Design and Experimental Methods

The device used in the development of the program had NACA 0030 symmetric airfoil pillars with no camber and a 30% thickness-to-cord ratio for the pillar region (Figure 1b). The airfoils were approximately 90 µm in length and 27 µm in thickness. The airfoils had a negative 15° angle-of-attack (α) from the bulk of the flow. Figure 1c shows the entire DLD device. The gaps (G) between the airfoil pillars were 40 µm with a row shift (Δ) of 8.4 µm. In addition, there was a filter region preceding the main displacement region with cylindrical pillars that had a decreasing gap size from 50 to 35 µm. A flow stabilizing region was located between the main array and the inlets of the device to reduce secondary flow generation before particle injection.

The SU-8 master molds were fabricated on a silicon wafer using a lithography tool. The DLD devices were then fabricated using a polydimethylsiloxane (PDMS) molding process. The cured PDMS was peeled off, and holes were punched for fluidic interconnects. Details of the procedure used to manufacture the devices can be found in our previous works [3,9,14,15].

Particles injection through the DLD was achieved with the use of three mechanical syringe pumps (kDScientific KDS-200: KD Scientific Inc. 84 October Hill Road Holliston, MA 01746, New Era NE-1000X: New Era Pump Systems Inc. 138 Toledo St. Farmingdale, NY 11735). The overall flow rate was controlled by the ratio of the three pumps [14]. Once the flow was stabilized, polystyrene particles of 10, 15, or 20 µm diameter were injected into the flow. For the development of the cell detection program using computer vision, only videos with the smallest CTCs (10 µm particles) were utilized, with the assumption that if the model can detect a particle of 10 μm, it shall be able to detect larger particles. It has been shown by Hou et al. [16] that the results of such particles are extrapolatable to a high degree of accuracy for actual biological samples. The combined overall flow rate through the device per the ratio of the syringe pumps ranged from 0.5 to 4.0 mL/min. A high-speed camera (Phantom Mira 310) was mounted to an optical microscope (Nikon Eclipse Ci) [9] to record the experiments. The frame rate of the camera varied within 1000–10,000 frames per second, depending upon the flow rate injected into the DLD.

### 2.2. Program General Information

Python, specifically version 3.7, was chosen as the programming language due to the availability of existing libraries and other resources for data management, machine vision, and machine learning. The Python packages used in the development of the program were as follows: Imutils [17], MatplotLib [18], NumPy [19], OpenCV [20], pandas [21], PIMS [22], scikit-learn [23], SciPy [24], and Yellowbrick [25].

Twelve videos with 10 µm diameter particles were used for the analysis tool development. Each video was manually analyzed to compare the manual results to the automation tool results. In order to examine the total particle count and the particle distribution, the focus of the program was on the outlet channels of the device, as shown in Figure 1. The computational time for analysis by the program was also calculated. The computational time did not include any time that required user interaction; the timer was paused any time for user interaction that occurred in the middle of the computation. The initial testing was completed on a computer with an Intel i7-7700K processor clocked at 4.20 GHz with 16 GB of RAM; the total computational time is affected by the computer hardware specifications.

**Figure 2:**
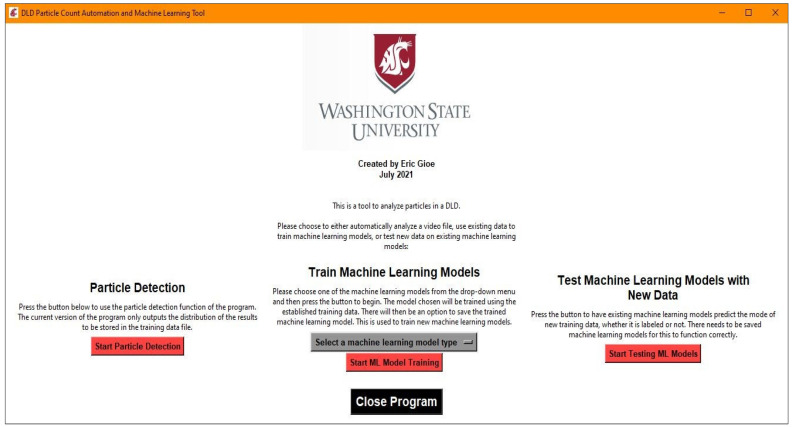
GUI used as the main window, which can launch one of three actions: the particle detection function, the machine learning model training function, and the function to test the existing machine learning models, if any are saved.

It was decided to create an all-inclusive executable that contained all of the resources for the program to avoid the user managing proper installations of Python and the appropriate packages and versions. To do this in a clean manner, a graphics user interface (GUI) was created using Tkinter, a native Python package. An example of the main menu GUI can be seen in Figure 2. The three major functions available for use are particle detection, machine learning model training, and testing existing machine learning models with new data. The particle detection functionality is the basis for the machine learning model training, since it is used to create a dataset that can be labeled for use in model training. Once an acceptable model is saved, it can be tested later on new data, but this will not be discussed in detail due to the similarity to the testing that is completed in the process of training machine learning models.

### 2.3. Machine Vision—Particle Detection

The analysis automation tool’s particle detection portion has the following major functions: automatic frame rotation, outlet channel detection, outlet channel wall detection, and particle detection with repeat detection prevention.

#### 2.3.1. Automatic Frame Rotation

The program was designed with the outlet channels horizontal in the observation window, or zero degrees in the unit circle. In those cases where the orientation of the DLD in the frame was not within a certain horizontal tolerance, the program would automatically rotate and crop the frame to maintain the outlet channels at a horizontal. This was accomplished through the use of probabilistic Hough transforms.

Similar to the outlet channel detection, the Hough transform output a series of coordinates that represented lines. The angle of these lines was calculated from the horizontal and then averaged. The frame was then rotated through Imutils’ [17] *rotate*_*bound* function to quickly rotate and crop the image about its center. The Hough transform was then repeated to confirm that the image after the rotation was within a certain tolerance. If the tolerance was not met, the process was repeated until the rotation converged into the tolerance; otherwise, it was stopped after a set number of repetitions. The total angle needed to rotate the frame was stored for further use. If the angle to rotate the frame was greater than twice the first value for rotation, the first rotation multiplied by a factor of 1.5 was used instead.

#### 2.3.2. Automatic Outlet Channel Detection

The automatic outlet channel detection was created due to a difference in practices when capturing data between the different researchers. This problem could have been solved through a standard procedure when recording data or with a fixture that guided the operator to the ideal recording location. However, it was decided to add some form of automated correction to the program.

**Figure 3:**
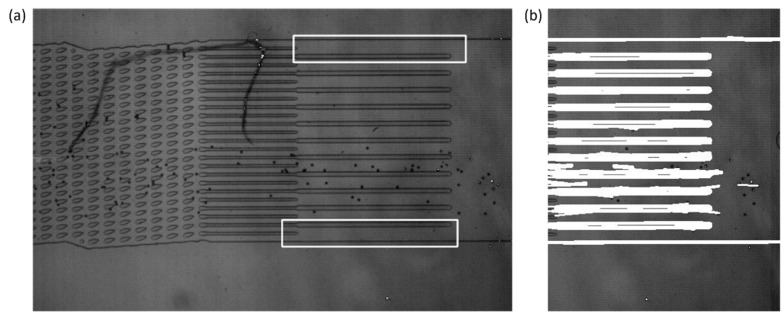
(**a**) The results of the template matching process on the original video frame. The location of the top and bottom templates is indicated by the white outline. The top and bottom templates’ most likely location is given by the *minMaxLoc* function when it is run on the output of the *matchTemplate* function. The left and right bounds of the observation window are calculated by averaging the results of the template matching. (**b**) Hough transform results where the white represents lines that were detected by the function. The row values that are the upper-most and lowest of the lines detected are used for the upper and lower bounds of the observation window, respectively.

The outlet channel detection relies upon OpenCV’s *matchTemplate* function to per-form a template matching technique to detect the outlet channels. Two templates were saved into the system for the DLD device that was prevalent in the development, one for the upper bound of the outlet channels and another for the lower bound of the outlet channels. The *matchTemplate* function would then output the upper-left corner of the matched location in conjunction with the use of the *minMaxLoc* function. An example of template matching results is demonstrated by Figure 3. These locations were used to determine the left and right bounds of the observation window by averaging the locations of the templates. The frame must be rotated to the horizontal prior to the template matching function, since the template matching function is sensitive to changes in rotation angle.

The upper and lower bounds of the observation window were found with the use of probabilistic Hough transforms, which were also a function in the OpenCV package. Based on the parameter input into the function, it output coordinates for lines found within the frame, based on the left and right bounds from the results of the template matching. From this, those lines were filtered for the upper-most and lowest lines detected, with the aim that it would detect the outermost walls of the outlet channels. The bounds of the observation window were stored for further use throughout the program.

#### 2.3.3. Outlet Channel Wall Detection

**Figure 4:**
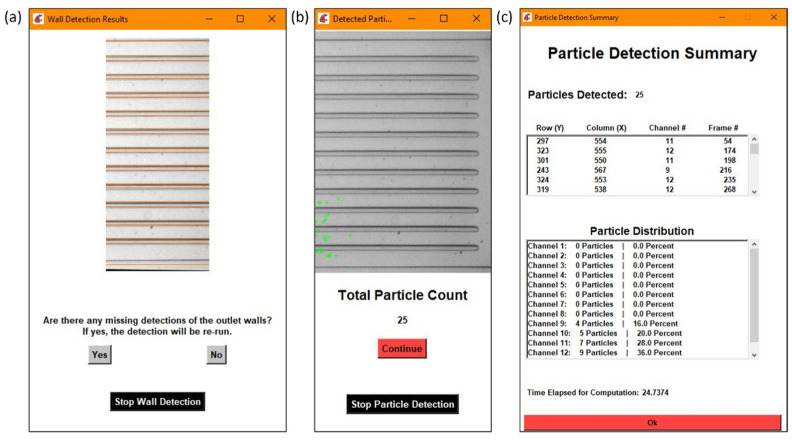
Each section is an example of the following: (**a**) wall detection results, (**b**) particle detection results, (**c**) a detailed summary of the particle detection results with the particle distribution calculation.

In order to properly determine the distribution of the particles in the outlet channels, the outlet channels themselves had to be defined by the program. The outlet channels were obtained through a combination of edge detection methods developed by Canny [26] and the Hough transforms. Canny edge detection resulted in a binary image containing those regions that were considered edges, or areas of higher contrast, and would typically represent the walls of the outlet channels if looking at the observation window calculated from previous steps. Those rows that were considered edges were then combined with a list of the rows that were considered lines by the Hough transform. All of the rows were then filtered through by grouping consecutive rows together and breaking the group when there was a significant gap. A row value was considered a wall when it had the greatest number of reported values in the list and a gap in reported values existed between it and the next grouping of values. An example of the output for the wall detection is demonstrated in Figure 4a.

#### 2.3.4. Particle Detection

**Figure 5:**
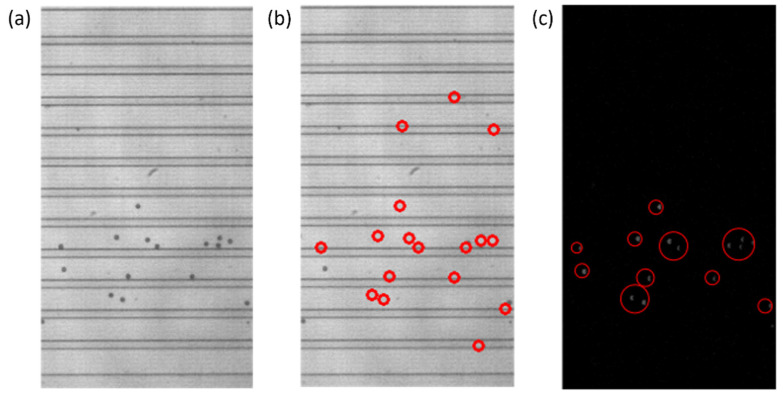
Steps of the particle detection process that include creating a background image for background subtraction and the intermediate steps of consecutive video frame comparison. (**a**) A zeroth frame of a video that contains particles. The image cannot be used as a generic background without modification due to the presence of particles. (**b**) The same zeroth frame of the video with the particles replaced with the average row value. Regions outlined by the red circles represent those particles that were removed from the newly created background image. (**c**) The intermediate step after the comparison or subtraction of consecutive video frames and removal of negative values, before the *SimpleBlobDetector* function is run. The gray blobs, outlined by the red circles, are particles that will be detected by the *SimpleBlobDetector* and will be compared to the list of previously detected particles.

The particle detection portion of the program compared two consecutive video frames to detect the particles in the flow. For reliable particle detection, the background subtraction method has been implemented. The background, consisting of the walls of the DLD, had to be isolated from any particles in the flow. Usually, this could be achieved by using a blank background image, but this is not easily achieved through various users with different practices when recording videos. The background had to be constructed from the video itself, with the “zeroth” or the very first frame of the video considered to be the most likely frame to contain solely the background. OpenCV’s *SimpleBlobDetector* function was run on the zeroth frame of the video to determine if particles were detected. If the zeroth frame was absent of particles, it was used for the background as is; those regions that were detected as particles were replaced with the average of each row the value was located in. This would replace any pixels that were particles with what should be the background brightness levels of the image. An example of this is presented in Figure 5.

**Figure 6:**
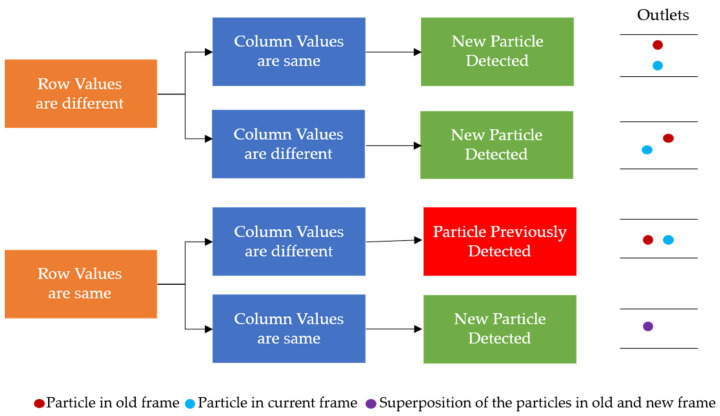
The rules for determining the repeat particle detection in consecutive frames based on the coordinates of the particles detected.

Once the background was subtracted from each of the two consecutive frames, the two frames were subtracted from each other, to only leave those values that would be particles in the compared values. With the subtraction, those negative values were replaced with zeros, and any resulting non-zero values should represent particles, as represented by the gray blobs in Figure 5c. The *SimpleBlobDetector* function was run on the resulting values to determine what was actually a particle, with filtering parameters such as the area and circularity used along with the thresholding capabilities of the function. The analysis in OpenCV itself provides no limitation on the detection of the cell concentration that is practical for the application of cell separation. In order to prevent repeat detection, a system of checks was implemented in the particle detection function, with some simple assumptions: particles will be flowing from left to right and never in the opposite direction, any particles that stop moving will be filtered out by the subtraction method, and the operator set an appropriate frame rate for the flow rate that was set for the system. In this work, to determine the state of the detected particles, two consecutive frames are analyzed, and the locations of the centroids of the particles are stored and compared. During comparison, if the row–column coordinates of the centroids of the new frame match with the respective coordinates of the centroids of the previous frame, they are stored in one array. Otherwise, if the centroids are different they are stored in another array. The row and column values of the particles of the two arrays are then compared with the previous frame, and only the new particles that are detected are stored. This yields three different scenarios for the new particle detection: (i) If the rows are found to be different and outside the tolerance of one pixel, then it is considered to be a new particle detection. (ii) If the columns values are different and at the same time if the row value are found to be outside one pixel, then it is considered to be a new particle. (iii) If the rows and columns of the centroid in the new frame are found to be same as those of the previous frame, then it is considered to be a new particle, since the particle will never stay in the same flow due to the motion of the fluid. These new particles are stored in an array and if the array of the new particle has any repeated values it is removed. By using the “unique” and “delete” functions of the open source python library “numpy”, the repeated particles are deleted and only the unique positions are kept. The rules for determining if a particle was already detected or not can be seen in Figure 6. An example of the particles detected in the observation window is displayed in Figure 4b.

In order to determine the outlet distribution, the coordinates of the detected particles were compared to the row values of the outlet channel walls. Each of the particles was assigned a channel and the coordinates were placed in a data storage matrix. Then, the percentage of particles in each outlet out of the total was calculated. Figure 4c shows a detailed summary including the coordinates of the center of each particle and the frame number it was detected in, along with the particle distribution.

### 2.4. Machine Learning—DLD Mode Prediction

The additional functionality of the automation tool is the DLD mode prediction for the inputs to the system. In DLD, particles are separated based on their size for a specific flow configuration. Consequently, 10, 15, and 20 μm particles were used to predict the different separation regimes. The largest cell will undergo bumped mode, the intermediate cell will undergo mixed mode, and the smallest cell will undergo zigzag mode (Figure 1a). The experiments conducted utilized 10, 15, and 20 μm particles to obtain the separation characteristics for each type of cell at different conditions. The result of the experiments was then used to train a machine learning model. The machine learning feature inputs for the DLD mode prediction included the flow rate, the particle size, and the particle distribution. The tool was created with three machine learning models for comparison, along with a method of testing saved machine learning models with newly acquired data. The default machine learning models built into the system were the complement naïve Bayes (CNB), K-nearest neighbors (KNN), and support vector machines radial basis function (SVM RBF) kernel. The three models were built in similar ways. All of them utilized a stratified k-fold cross validation method for the training and testing the data, where the data splits conserve the proportionality of the data as much as possible. The data were split into five partitions, with one section preserved as the testing data while the other four were used as the training data. The five splits were rotated so that each split become the testing data.

The data used to train the machine learning models consisted of 66 runs based on the results generated by the particle detection function. For a dataset to train machine learning models, this is rather small. Owing to the fact that there is a small number of features that control the flow physics of DLD and the separation of cells, a small dataset can be expected to give a generalized model. Conducting DLD experiments for data collection, manual analysis of the results, and preprocessing of the data to feed into the algorithms for training are currently the biggest hurdles in working with a larger sample. From the computer vision and the machine learning perspectives, the algorithms are scalable for practical applications in the field of particle/cell separation and do not possess any challenge while working with a larger sample size, although computation times are expected to increase.

After splitting the data, the machine learning models were then trained on each of the training and testing data splits, with the highest accuracy data split being the model that was saved. The only difference is that the KNN model has the additional step of testing a range of a minimum number of neighbors, from 2 to 55, for each of the data splits. The smallest number of neighbors with the highest accuracy was saved for its respective data split. The results for each of the data splits were compared, with the model with the highest accuracy saved. The results of the highest accuracy machine learning models were displayed through a classification report and a confusion matrix with the Yellowbrick package.

## 3. Results and Discussion

### 3.1. Machine Vision—Particle Detection

**Figure 7:**
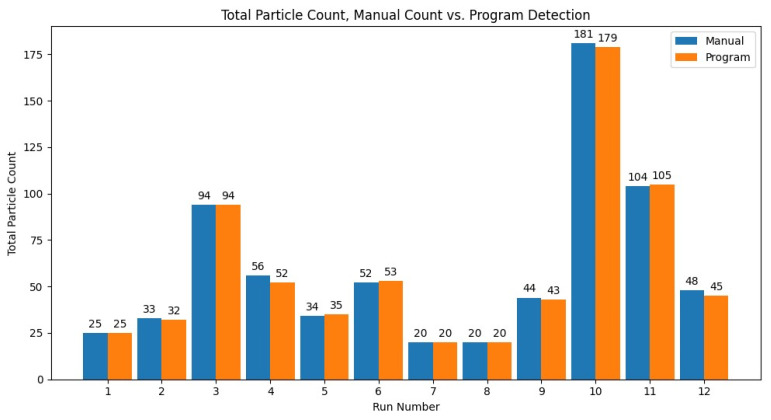
The total particle count of the program versus the manual count for the 12 manually analyzed videos (run number) with 10 µm diameter.

To test the accuracy of the particle detection function of the program, the results of 12 video files were compared between the program and manual analysis. Since the manual analysis can take upwards of a few hours, it limited the number of files for manual analysis. The results for the total particle count for the 12 runs compared to the manual analysis can be seen in Figure 7. The percent error was averaged across the 12 runs, with an average error of 2.14% and sample standard deviation of 2.42, or an overall total particle count accuracy of 97.86%.

There was a weak inverse linear correlation of −0.122 between the total particle count and the percent error compared to the manual count. This weak inverse correlation indicated there was not a significant trend between a larger particle count causing larger percent errors. The program also calculated the particle distribution, or the number of particles per channel. Those results were compared to the manually determined particle distribution. The results of the particle distribution of the program can be seen in Table 1. Those outlets that were colored yellow, orange, or red indicate instances where the total number of particles was outside of a five-particle window from the actual distribution reported by the manual analysis. There were 4/12 runs that had each outlet within a one-particle tolerance of the manual analysis. Otherwise, the other 8/12 had some amount of error greater than two to five particles compared to the manual analysis. For a few of the runs, the assignment of the outlet channel values was skewed upward by one row, or in other words, there was a visible shift in the values that were assigned to each outlet. An example of this is in run 9.

Additionally, the computational time without user interaction was recorded for each of the 12 runs. The average time to complete the computation of the program was 25.274 s, with a sample standard deviation of 0.724 s. The computational time will vary with differences in the specifications of computer hardware.

In terms of meeting the goal set out of providing an accurate particle detection program, the results show that for the total particle count, the system developed does this with high accuracy. It is not even more accurate due to the nature of the *SimpleBlob-Detector* function, which used some level of thresholding that could exclude some of the particles based on the brightness in the field. A different system could be developed, but this was sufficient for the current level of development. However, the particle distribution calculation needs improvement. With some investigation it was observed that some of the issues stemmed from the wall outlet determination, which was normally verified by the user, but was not properly assigning some of the channels. In some cases, the top of the bottom of the frame was cut off after the rotation, so it is not as robust as needed with determining the outlet walls. This could also be from the automatic observation window determination, which could incorrectly skew the observation window and remove the outermost features of the DLD, depending upon the quality of the recording.

### 3.2. Machine Learning—DLD Mode Prediction

**Figure 8:**
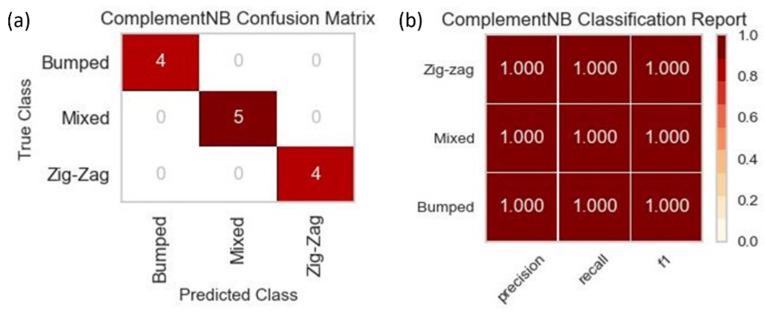
(**a**) The confusion matrix for the CNB machine learning training on one of the five data splits with a total dataset size of 66. The diagonal values represent the correct prediction of the DLD mode by the machine learning model, while the off-diagonal values represent those predictions that were incorrect. (**b**) The classification report for the CNB machine learning training that displays the precision, recall, and f1-score for each of the DLD modes.

Due to the size of the dataset used to train all three of the machine learning models, the results for predicting the three DLD modes were not drastically different across the three. The results for one of the three models, since they were identical in their visual forms, can be seen in Figure 8. The accuracy was at 100%, which meant that the precision, recall, and f1-score were at their highest possible values of 1. The similarity in results shared across the three models was also due to the nature of the classification with only three possible outcomes. Although the accuracy for all three models was 100% based on the training with the small dataset, a more extensive dataset needs to be implemented with more diverse data across all available features to prevent potential over-fitting.

In addition, the trained machine learning models may struggle to predict the result accurately if they are presented with a drastically different particle distribution with the flow rate and particle size. Due to the nature of creating the dataset from the analysis automation tool, there is room for growing the dataset size with more experimentation.

## 4. Conclusions

DLD is a microfluidic method that has been widely implemented for various bioparticle separations, such as circulating tumor cells from blood components. A properly designed DLD for high-throughput flow offers rapid particle separation, but requires the ability to effectively analyze a vast amount of data. In this work, we have developed a software tool that automated the process of observing and characterizing the flow of particles through high-throughput experimental DLD devices to minimize the time required for analysis.

The analysis automation tool can provide rapid results by reducing the analysis time of DLD videos in an easy-to-use manner. For the basis of the tool, a reliable method of particle detection was created with influence from works in automation of other microfluidic processes. This was accomplished through Python and its available packages in machine vision techniques such as probabilistic Hough transforms, Canny edge detection, and template matching, and existing tools such as OpenCV’s *SimpleBlobDetector*. The analysis automation tool was able to detect particles consistently and with a high accuracy. The final version of the program had an overall particle detection accuracy of 97.86% while completing the computation in an average time of 25.274 s.

Three machine learning techniques, CNB, KNN, and SVM RBF kernel, were implemented and compared in the determination of the DLD mode. Since the dataset used was rather small the accuracy for the three models was 100% for all of them. If the dataset were larger, there would be less of a chance that the machine learning models would over-fit on the data used to train them. More data need to be acquired, analyzed, and labeled to have a larger dataset when re-training the machine learning models.

Additional functions can be added that enhance the capabilities of the analysis automation tool. An example is identifying circulating tumor cells in the flow to determine if they were properly isolated by the device, which could be accomplished through machine learning by training the algorithm on images of circulating tumor cells and other blood components. The developed method could reduce human errors and save time for the separation verification.

## Figures and Tables

**Table 1:** The particle distribution throughout the outlets across the twelve runs. The blank spaces represent zeros. The absolute difference of the program versus manual count is color-coded by the following rules: ■≤±2%, ■≤±5%, ■≤±8%, ■≤±10%, ■>±10%.

Outlet #\Run	1	2	3	4	5	6	7	8	9	10	11	12
1			1			1						1
2						14	3				6	4
3					6	1	2				14	7
4				4	9	6	2			53	36	6
5			23	14	9	10	9			41	10	8
6			13	5	3	4	1			14	10	7
7		3	11	11	6	10	2			30	8	6
8		3	17	13	1	6	1	3	3	24	12	5
9	4		16	4	1	1		5	14	6	5	
10	5	17	10	1				8	16	4	3	1
11	7	7	2					1	7	1		
12	9	2	1					3	3	1	1	
Total	25	32	94	52	35	53	20	20	43	179	105	45

## Data Availability

The dataset generated for this paper can be found in the resources for the analysis tool located at https://gitlab.com/eric.gioe/particle-detection-automation-tool (accessed on 1 December 2021).

## Abbreviations

The following abbreviations are used in this manuscript:

DLD	Deterministic lateral displacement
CNB	Complement naïve Bayes
KNN	K-nearest neighbors
SVM RBF	Support vector machines radial basis function
OpenCV	Open source computer vision library
PIMS	Python image sequence
*Re*	Reynolds number
